# Five Different Types of Framing Effects in Medical Situation: A Preliminary Exploration

**DOI:** 10.5812/ircmj.8469

**Published:** 2013-02-05

**Authors:** Jiaxi Peng, Hongzheng Li, Danmin Miao, Xi Feng, Wei Xiao

**Affiliations:** 1 Department of Psychology, Fourth Military Medical University, Xi'an, China; 2 Mental Health Center, 303 Hospital, Nanning, China

**Keywords:** Framing Effect, Medical Decision Making

## Abstract

**Background:**

Considerable reports concerned the framing effect in medical situations. But quite few of them noticed to explore the differences among the various kinds of framing effects.

**Objectives:**

In the present study, five different types of framing effects were examined and the effect sizes of them were compared.

**Materials and Methods:**

Medical decision making problems concerning medicine effect evaluation, patient's compliance, treatment and doctor options selection were established. All the problems were described in both positive and negative frames. 500 undergraduates as participants were randomly divided into ten groups. Participants from each group were asked to finish one decision making task.

**Results:**

All the frames that were examined leaded to significant framing effects: When the Asia Disease Problem was described in a positive frame, the participants preferred the conservative frame than the risky one, while if in a negative frame, the preference reversed (P < 0.01). If the drug effect was described as “of 100 patients taking this kind of medicine, 70 patients became better”, people tended to make more positive evaluations, compared with described as “of 100 patients taking this kind of medicine, 30 patients didn’t become better” (P < 0.01). Doctors’ advices were respectively described in a baneful or beneficial frame and the former one resulted in a better compliance (P < 0.05). If treatment options were described with a survival rate, people tended to choose risky option, while if described with a mortality rate, people tended to choose conservative option (P < 0.05). The number sized framing effect was also tested to be significant (P < 0.01). The five types of framing effects were small to big in effect size.

**Conclusions:**

Medical decision making can be affected by frame descriptions. Attentions should be paid on the standardization of description in medical practice.

## 1. Background

Framing effect refers to when the same problem was presented using different representations of information, people made significant changes to their decisions or even reversed their decisions (1). The Asian Disease Problem is a classic example: Imagine that the United States is preparing for an outbreak of an unusual Asian disease that is expected to kill 600 people. Two alternative programs have been proposed to combat the disease. Scientific estimates of the consequences of the programs are figured out. Positive frame: If Program A is adopted, 200 people will be saved; If Program B is adopted, there is 1/3 probability that all 600 people will be saved and 2/3 probability that no one will be saved. Negative frame: If Program C is adopted, 400 people will die; If Program D is adopted, there is 1/3 probability that all none will die, and 2/3 probability that all people will die. Since A & C and B & D were logically equivalent, there should be no difference in preference for people. Also, there should be no shift of preferences from risk seeking to risk avoiding simply because of how the problem is described. However, Tversky and Kahneman reported in their study that 71% of the participants would choose A rather than B in a positive frame, while 72 % of the participants would choose D rather than C in a negative frame (1).

Considerable studies followed suggested that framing effect was a wide spread and robust phenomenon, and steadily existed in various fields of decision making problems, like economy, lifesaving, resource allocation and management (2-8). While some studies also found that there were certain situations particularities in different frames. Levin, Schneider and Gaeth identified three types of framing tasks as: risky-choice framing, for example, Asia Disease Problem; attribute framing, like a pound of beef can be described as “80 lean” or “20% fat”, may significantly influence people’s evaluation; goal framing which refers to the description of the benefit brought from actions or the aftermath of no actions may significantly influence people’s motivation (9). Levin, et al. found the Structure-specificity among the three kinds of frames (10). Frisch suggested that the candidate options were not always required equivalent (11). For example the widely used medical decision making study material provided the following situation: The decision maker should make a choice between having surgery or radiation therapy. The surgical treatment had a lower treatment survival rate, but a relatively higher 5-year survival rate; while the radiation-therapy treatment had a higher treatment survival rate, but a lower 5-year survival rate. The problem could be presented in a positive (survival rate) or negative (mortality rate) frame which might also result in the risk preference shift, though the effects of surgery and radiation therapy were not equivalent. In a recent study conducted by Wong and Kwong, a new kind of framing namely number-size framing was found, which reflected the law of diminishing marginal utility (12). Specifically speaking, people are sensitive to the change from 1 to 2, but not sensitive to the change from 101 to 102.

In medical practice, both doctors and patients are often faced with a number of decision-making problems which can also be affected by framing. For example, Bigman et al. reported that describing the effect of a human papillomavirus (HPV) prophylactic to some participants as being 70% effective (positive frame) and to others as being 30% ineffective (negative frame) produced different results. Even though there was no actual difference between the two frames, the participants who received the positive frame believed that it had a better prophylactic effect and were more willing to receive vaccine (13). In the present study, we examined the five types of framing effects that have emerged, and focused on the differences in effect size among the five framings.

## 2. Objectives

In the present study, five different types of framing effects were examined and the effect sizes of them were compared.

## 3. Materials and Methods

### 3.1. Participants

500 male undergraduates, with a mean age of 19.46 years (SD = 2.04), participated for extra course credits. Of the 500 questionnaires distributed, 500 were returned. Among these, all were usable responses, resulting in a valid response rate of 100%. The participants were randomly divided into 10 groups and participants from each group only answered one decision making problem.

### 3.2. Methods

Based on the distinction by Levin, etc, Frisch and Wong etc, five medical decision making problems were adopted, and all the problems were described in both positive and negative frames. The responses were made on a 6 - point Likert scale ranging from 1 (very tend to option A) to 6 (very tend to option B). Similar to a simple dichotomous scale where a participant would choose one of the two options, participants using a 6 point-scale must also favor one procedure over the other since there is no mid-point. In addition, the 6-point scale allowed us to determine the strength of the choice preference (14).

## 4. Results

As shown in Table 1, the results revealed that if drug effect was described respectively in a therapeutically effective rate or ineffective rate, the participants gave the former a more positive evaluation (t = 3.34, P = 0.01). As shown in Table 2 that if the doctor’ advise was respectively described in the benefit of compliance and the aftermath of violation, the former one lead to a better compliance (t = 2.14, P = 0.035).

Table 3 presented the classic Asia Disease Problem which we used as an example of risky choice framing of options equivalent. The results revealed that in a positive frame, people tend to be risk seeking, while in a negative frame, people tend to be risk avoiding. The framing effect was significant (t = -2.72, P = 0.007). Table 4 described the risky choice framing effect when the candidate options were not equivalent. As the results revealed, if treatment options were described in survival rates, people more preferred the risky option, compared with described in mortality rates (t = 2.09, P = 0.039). Table 5 described the effect of number size framing. The results revealed that the number size framing effect was significant in medical situation (t = -7.5, P < 0.001).

**Table 1:** The Attribute Framing Effect

Questionnaire	Mean Response ^a^
Positive: 100 patients taking one kind of medicine, 70 patients became better. How would you evaluate the drug effect?	4.36 ± 1.17
Negative: 100 patients taking one kind of medicine, 30 patients didn’t become better. How would you evaluate the drug effect?	3.26 ± 2.01

^a^Response was made from “1” means “very bad” to “6” means “very good

**Table 2:** The Goal Framing Effect

Questionnaire	Mean response ^a^
Positive: Doctor tells you that although you particularly like to eat bacon, if you stop eating, your body cholesterol content would be significantly reduced, and thus the possibility of suffering from cardiovascular disease would be greatly reduced. Would you continue to eat bacon?	2.56 ± 1.23
Negative: Doctors tells you that although you particularly like to eat bacon, if you continue to eat, your body cholesterol content will significantly rise, and thus the possibility of suffering from cardiovascular disease would be greatly increased. Would you continue to eat bacon?	2.10 ± 0.89

^a^Response was made from “1” means “surely stop eating” to “6” means “surely continue eating

**Table 3:** The Risky Choice Framing Effect (Options Equivalent)

Questionnaire	Mean Response ^a^
Positive: There are 600 critically ill patients in one hospital. Two rescue programs are under consideration. If program A is adopted, 200 patients will be saved; If program B is adopted, there is a 1\3 chance that all patients will be saved and there is 2/3 chance that none will be saved. Which program will you choose?	2.94 ± 1.46
Negative: There are 600 critically ill patients in one hospital. Two rescue programs are under consideration. If program A is adopted, 400 patients will die; If program B is adopted, there is a 1/3 chance that none of the patients will die and there is 2/3 chance that all will die Which program will you choose?	3.84 ± 1.82

^a^Response was made from “1” means “surely choose A” to “6” means “surely choose B”

**Table 4:** The Risky Choice Framing Effect (Options Not Equivalent)

Questionnaire	Mean Response ^a^
Positive: Imagine one of your relatives was diagnosed with a cancer that must be treated. His choices are as follows: Surgery: Of 100 people having surgery, 50 live through the operation, and 40 are alive at the end of five years. Radiation therapy: Of 100 people having radiation therapy, all live through the treatment, and 20 are alive at the end of five years. Which treatment would you advise him to choose?	3.22 ± 1.40
Negative: Imagine one of your relatives was diagnosed with a cancer that must be treated. His choices are as follows: Surgery: Of 100 people having surgery, 50 die because of the operation and 10 of the 50 survivals die by the end of five years. Radiation therapy: Of 100 people having radiation therapy, none die during the treatment, and 80 die by the end of five years. Which treatment would you advise him to choose?	2.68 ± 1.17

^a^Response was made from “1” means “surely choose radiation therapy” to “6” means “surely choose surgery”

**Table 5:** The Number Size Framing Effect

Questionnaire	Mean Response ^a^
Framing 1: Eye surgery may lead to two potential sequelas: one is a minor decline of visual acuity and the other is keratitis. Imagine you will take this kind of surgery and two doctors’ medical records revealed that: Doctor A: Of 200 patients, 191 were not found postoperative visual acuity declined but 3 suffered from keratitis; Doctor B: Of 200 patients, 198 were not found postoperative visual acuity declined but 10 suffered from keratitis. Which doctor you will choose?	2.18 ± 1.22
Framing 2: Eye surgery may lead to two potential sequelas: one is a minor decline of visual acuity and the other is keratitis. Imagine you will take this kind of surgery and two doctors’ medical records revealed that: Doctor A: Of 200 patients, 197 didn’t suffer from keratitis but 9 were found visual acuity declined. Doctor B: Of 200 patients, 190 didn’t suffer from keratitis but 2 were found visual acuity declined. Which doctor you will choose?	4.14 ± 1.39

^a^Response was made from “1” means “surely choose doctor A” to “6” means “surely choose doctor B”

**Figure 1:**
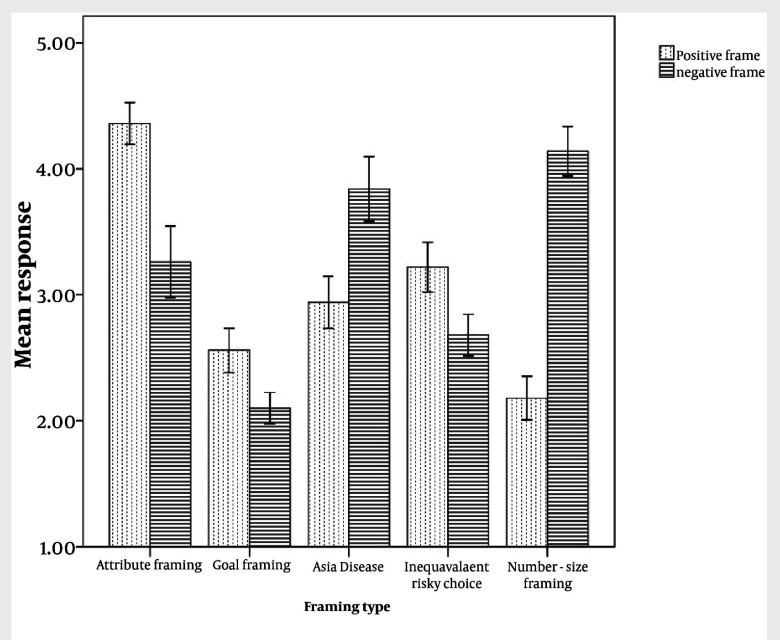
The Five Types of Framing Effects

Figure 1 presented the five framing effects. Then we analyzed the effect size of the five types of framing effect. Effect size (ES) is a name given to a family of indices that measure the magnitude of a treatment effect. Unlike significance tests, these indices are independent of sample size. The bigger effect size demonstrates the stronger effect or phenomenon. In the current study, the index of Cohen d was chosen, which could be expressed in formulas as d = (-)/σ_pooled_. σ_pooled_ is the pooled standard deviation of two comparing sets of data. Table 6 presented the Cohen d of the five types of framing effects. According to Cohen’s study, the usual standards of small, medium and big effect sizes presenting in the Cohen d were respectively 0.4, 0.5, and 0.8 (15). Thus the goal framing effect reflected a small effect size, and attribute and risky choice framing effects were medium effect size and number size framing effect appeared a big effect size.

**Table 6:** The Effect Sizes of the Five Types of Framing Effects

Framing Type	Attribute	Goal	Risky Choice (Equivalent)	Risky Choice (Not Equivalent)	Number Size
**Cohen d**	0.64	0.40	0.52	0.42	1.21

## 5. Discussions

The present study examined the five types of framing effects that have emerged in medical situation and found that framing effect was a robust and steady phenomenon. In an attribute frame, when the drug effect was described in a loss frame, people made significantly negative evaluation compared with described in a gain frame. This is consisting with prior research: A pound of beef can be described as “80 lean” or “20% fat”, and people give the former one better evaluation (10). In a goal framing: The doctors’ advices were respectively described as the benefit brought from compliance and the aftermath of aviation. The participants complied with the later one more readily. In the classical Asia disease problem, people become conservative in a positive frame but risky in a negative frame. In the number sized framing effect, the change of the description also lead to a revision of decision making. The three types of framing effect can all be explained based on the Prospect Theory. According the model, people’s cognition of gains or losses is relative to the reference point, rather than from the absolute level of wealth. Gains and losses are evaluated differently due to the shape of value function which is concave in the gain area and convex in the loss area, reflecting the law of diminishing marginal utility. The loss function is steeper than the gain function, implying that decision makers are more sensitive to losses than gains (16, 17). In a goal framing, “Increase of the probability of catching a disease” can be regarded as a loss, while “decrease of the probability of catching a disease” can be regarded as a gain. So people complied with doctor’s advices more readily when these advices were described as the aftermath of no cooperation. In a number size framing, people are more sensitive to the change from 3 to 10, compared with the change from 190 to 197. So the former description can affect people’s decision. In the classical Asia Disease Problem, though “200 patients can be saved” and “400 patients will die” are logically equivalent, but the two descriptions are understood differently since they are separately above or under the reference point. Due to the marginal utility, the psychological value of saving 200 patients is bigger than that of 1/3 probability of saving 600, thus decision maker are conservative in positive frames. In the same way, the hurt of 2/3 probability of losing 600 lives is not as much as that of losing 400 lives for sure, then decision makers tend to be risky in negative frames. In the risk choice framing with not equivalent options: When people were informed the survival probability of treatment prospects, they were more inclined to choose the radiation therapy treatment with the short-term benefit and long-term loss than mortality. It can be explained as: When participants were informed as survival rate they regarded it as an opportunity rather than a threat; but when they were given mortality information, they thought of it as a threat more than an opportunity. Numerous studies have indicated that when the decision makers perceive opportunities more than threats, they are more inclined to take risks; in contrast, when they perceive more threats than opportunities, they are more conservative (18, 19). In addition, people will consider the future only in a safe environment. If they feel they are threatened, their attentions will be more focused on the present.

The effect sizes of the five types of framing effects were not the same, the values of Cohen d ranging from 0.40 to 1.21. The number size framing rustles in the strongest effect. The results of the present study suggest that medical decision making can be affected by frame descriptions. Attentions should be paid on the standardization of description in medical practice.
